# Headspace/GC–MS Analysis and Investigation of Antibacterial, Antioxidant and Cytotoxic Activity of Essential Oils and Hydrolates from *Rosmarinus officinalis* L. and *Lavandula angustifolia* Miller

**DOI:** 10.3390/foods10081768

**Published:** 2021-07-30

**Authors:** Stefania Garzoli, Valentina Laghezza Masci, Sara Franceschi, Antonio Tiezzi, Pierluigi Giacomello, Elisa Ovidi

**Affiliations:** 1 Department of Drug Chemistry and Technology, Sapienza University, 00185 Rome, Italy; pierluigi.giacomello@uniroma1.it; 2 Department for the Innovation in Biological, Agrofood and Forestal Systems, Tuscia University, 01100 Viterbo, Italy; laghezzamasci@unitus.it (V.L.M.); antoniot@unitus.it (A.T.); eovidi@unitus.it (E.O.); 3 Department of Economics and Statistics, University of Siena, 53100 Siena, Italy; franceschi2@unisi.it

**Keywords:** *Lavandula angustifolia*, *Rosmarinus officinalis*, essential oils, hydrolates, chemical analysis, volatile compounds, biological activity

## Abstract

In this work, essential oils (EOs) and hydrolates (Hys) of *Rosmarinus officinalis* L. and *Lavandula angustifolia* Mill., grown in Tuscany (Italy), were studied to describe their chemical composition and biological activities. The aromatic profile of the EOs liquid phase was carried out by gas chromatography–mass spectrometry (GC–MS), while the volatile composition of vapor phase EOs and Hys was performed by headspace (HS)/GC–MS. The obtained results show that monoterpene hydrocarbons (71.5% and 89.5%) were the main compounds, followed by oxygenated monoterpenes (26.0% and 10.5%) in the liquid and vapor phase of *R. officinalis* EO, respectively. The oxygenated monoterpenes were the main components of *L. angustifolia* EO, reaching 86.9% in the liquid phase and 53.7% in the vapor phase. Regarding Hys, they consisted only of oxygenated monoterpenes, and 1,8-cineole (56.2%) and linalool (42.9%), were the main components of *R. officinalis* and *L. officinalis* Hys, respectively. Their cytotoxicity was investigated on an SHSY5Y neuroblastoma cell line by thiazolyl blue tetrazolium bromide (MTT) test, showing a notable effect of the EOs with a time-independent manner of activity and half maximal effective concentration (EC_50_) values quite similar for the two plant species (from 0.05% to 0.06% *v*/*v* for the three time points evaluated). A measurable activity of Hys was also obtained although with higher EC_50_ values. The antibacterial activity against *Escherichia coli* ATCC^®^ 25922, *Pseudomonas fluorescens* ATCC^®^ 13525, *Acinetobacter bohemicus* DSM 102855 as Gram-negative bacteria and *Kocuria marina* DSM 16420, *Bacillus cereus* ATCC^®^ 10876 as Gram-positive bacteria, was evaluated by the agar disk-diffusion method and the VPT (vapor phase test) to determinate the MIC (minimal inhibitory concentration) and the MBC (minimal bactericidal concentration) values. Both EOs possessed a high activity against all the bacterial strains with MIC values ranging from 0.19% to 3.13% *v*/*v*. Unlike EOs, Hys did not show an inhibition of the bacterial growth at the tested concentrations. Furthermore, antioxidant power was measured by 2,2′-azino-bis (3-ethylbenzothiazoline-6-sulfonic acid) diammonium salt-based (ABTS•+) and the 2,2-diphenyl-1-picrylhydrazyl (DPPH) assays, showing a remarkable ability to reduce radicals by both EOs; Hys were slightly less active. The findings highlighted that *R. officinalis* and *L. angustifolia* EOs and Hys have a chemical composition rich in bioactive molecules, which can exert different biological activities.

## 1. Introduction

Since historic times, spices and herbs have been used as food flavors, and their effects on human health are still being investigated to understand the roles of their chemical components [1,2]. *Rosmarinus officinalis* L. (also known as *Salvia rosmarinus* Schleid) and *Lavandula angustifolia* Mill. belong to the Lamiaceae family, which comprises different genera whose biological activities are used in traditional medicines all over the world [3,4,5,6]. Rosemary, native to the Mediterranean area, is a shrubby plant up to 1.8 m tall, erect or procumbent with a good aromatic scent due to the glandular hairs that emit volatile essential oils [7]. This plant is widely used in cosmetic preparations to protect from degradation and absorbing UV light, is used as a bactericidal and antifungal agent and furthermore, among others, was exploited in topical applications for wound healing, skin cancer and antimycotic properties [5,7]. Different uses of *R. officinalis* are known and its volatile essential oil (EO) and leaf extracts possess extensively investigated biological properties, such as antioxidant, anti-inflammatory, antiproliferative, anticancer, antiviral, antimicrobial, hepatoprotective, neuroprotective, nephroprotective, antiulcer and many others [8]. *R. officinalis* was investigated for its curative properties against some ailments caused by biochemical, chemical or biological agents as reviewed by Oliviera et al. [9], showing that this plant possesses beneficial effects and may be used to treat health problems. English or “true” lavender, the common names of *Lavandula angustifolia*, is one of the 39 species of the genus Lavandula to which belongs different hybrids [10]. Lavender EO production and quality is regulated by environmental and developmental conditions and temperature and flowering stage determine its chemical composition [11]. *L. angustifolia* EO is used in perfumery and cosmetics and its activity on the central nervous system, as a sedative, anxiolytic and antidepressant, was also evidenced [12,13,14]. Furthermore, biological activities of EOs from Lavandula genus, such as antifungal, antibacterial, antioxidant and anticancer effects, were reported [15,16,17,18,19,20]. EO chemical composition is highly complex and it can vary considerably depending on several factors, such as the cultivation area, environmental conditions, morphological characteristics and processing techniques of the plant [21,22,23]; moreover, the chemical composition influences the way in which EOs exert their antibacterial activity [24]. Terpene and sesquiterpene hydrocarbons, oxygenated or cyclic, are the main classes of compounds present in EOs, followed by aldehydes, ketones, alcohols, acids and esters [25]. In particular, thanks to their interesting physicochemical characteristics, Lamiaceae EOs were employed in the industrial and medical research sectors as natural products [26]. EOs obtained from *L. angustifolia* and *R. officinalis* grown in different countries such as China, Siria, India, Iran, Romania, Canada, Spain, France and others were investigated and linalool, borneol, linalyl acetate and 1,8-cineole as well as camphor, camphene and α-pinene resulted as predominant compounds, although in different proportions according to the vegetative stage and climatic conditions of the origin area [27,28]. There are far fewer studies concerning the volatile chemical composition of hydrolates (Hys), also known as hydrosols. They are aqueous solutions obtained as by-products of distillation [29] containing a certain number of bioactive molecules, although with marked quantitative and qualitative differences compared to EOs [30]. Their aroma can be more or less intense depending on their content of molecules that provide a potential biological effect, making the hydrolates useful for the food industry as preserving and/or aromatic agents [31]. In this study, for the first time, to better describe the vapor phase chemical profiles of Hys and EOs obtained from flowers and inflorescences of *R. officinalis* and inflorescences of *L. angustifolia* growing in Tuscany, we used the automated headspace sampler directly coupled with gas chromatography–mass spectrometry (HS/GC–MS) [32,33]. This sampling technique is conservative and non-destructive and does not require the use of solvent for the extraction process, thus avoiding a possible loss of components. The chemical composition of the liquid phase of EOs was also characterized by GC/MS and their antiproliferative, antibacterial and antioxidant activity was evaluated. Moreover, this is the first report revealing the biological activity of the vapor phase and the cytotoxic activity against a neuroblastoma cell line of *Lavandula angustifolia* and *Rosmarinus officinalis* Hys.

## 2. Materials and Methods

### 2.1. Materials

EOs and Hys from flowers and inflorescences of *R. officinalis* L. and inflorescences of *L. angustifolia* Mill. growing in Tuscany (San Donato in Poggio and Roccastrada, respectively), Italy and obtained by steam distillation, were directly provided by “èssenziale” Azienda Agricola, San Donato in Poggio (FI), Italy. The dates of collection of plants were: June 2020 for *R. officinalis* and July 2020 for *L. angustifolia.* Lysogeny Broth (LB) with agar, thiazolyl blue tetrazolium bromide (MTT), vinblastine sulfate, methanol, 2,2-diphenyl-1-picrylhydrazyl (DPPH), 6-hydroxy-2,5,7,8-tetramethylchroman-2-carboxylic acid (Trolox), 2,2′-azinobis (3-ethylbenzothiazoline-6-sulfonic acid) diammonium salt (ABTS) and potassium persulfate (K_2_S_2_O_8_) were from Merck (Darmstadt, Germany). Gentamicin sulfate was bought from Biochrom PAN-Bio-Tech GmbH (Aidenbach, Germany).

### 2.2. GC–FID and GC–MS Analysis

The GC–MS analyses were performed with a gas chromatograph equipped with a flame ionization detector (FID) and coupled to a mass spectrometer (MS), Perkin Elmer Clarus 500 model (Waltham, MA, USA). The GC capillary column was a Varian Factor Four VF-1 and helium served as a carrier gas at a flow rate of 1 mL/min. The injector temperature was 280 °C and the oven temperature program started from 60 up to 220 °C for 20 min at a rate of 6 °C min^−1^. For liquid injections, the solutions were prepared by diluting 1 μL of each EO with 1 mL of methanol and 1 μL of the sample was injected. MS operative conditions were: ionization voltage of 70 eV and acquisition mass range 40–450. Ion source and the connection parts temperature was 220 °C. The GC–TIC mass spectra were obtained by the TurboMass data analysis software (Perkin Elmer-Vers. 6.1.0). The identification of components was performed by matching their mass spectra with the spectrometer database of the NIST and Wiley libraries and comparison of their linear retention indices (LRIs) calculated against a mixture of *n*-alkanes (C8–C30). The relative average percentages of compounds were calculated by peak area normalization from GC–FID chromatograms without the use of an internal standard or correction factors. All analyses were conducted in triplicate.

### 2.3. HS/GC–FID and HS/GC–MS Analysis

To describe the vapor phase profile of EOs and Hys, a Perkin-Elmer Headspace (HS) Turbomatrix 40 (Waltham, MA, USA) autosampler connected to GC–MS was used [34,35]. About 1 mL of EO and 2 mL of Hy were placed separately in 20 mL vials sealed with headspace (PolyTetrafluoroethylene-PTFE)-coated silicone rubber septa and caps. The operative optimized conditions were: the sample was heated at 60 (EOs) and 80 °C (Hys) for 20 min thermostating time and an injection volume of about 10 mL (vapor phase) was sent to the capillary column of GC by a transfer line maintained at 200 °C. Quantification and identification of compounds was carried out by GC–FID and GC–MS analyses.

### 2.4. Cell Culturing

To assess the biological activity of the examined EOs and related Hys, human neuroblastoma SHSY5Y (ATCC^®^ CRL-2266™) cell lines were used. The cells were maintained in a 75 cm^2^ flask containing DMEM-F12 (Dulbecco’s modified Eagle’s medium: nutrient mixture F-12) culture medium supplemented with 10% of FBS (fetal bovine serum), 1% glutamine and 1% penicillin/streptomycin, and maintained at 37 °C with 5% CO_2_ and controlled humidity. Once the cells reached confluence, they were passed into new culture vessels in a 1:20 ratio and the medium was changed every 3 days.

### 2.5. Cytotoxicity Test (MTT)

The cell viability of SHSY5Y cells treated with *R. officinalis* and *L. angustifolia* EOs and Hys was evaluated by MTT assay. The mitochondrial dehydrogenase activity of the control and treated cells, which reflects their cell viability, was analyzed both in a dose- and time-dependent manner. A total of 2 × 10^4^ cells/well were seeded in a 96-well plate 24 h before being treated. EOs were dissolved in Dimethyl Sulfoxide (DMSO) (50% *v*/*v*). Twelve two-fold diluted concentrations were applied from 1 × 10^−1^% to 2 × 10^−4^% *v*/*v* for the EOs and from 50% to 1 × 10^−1^% *v*/*v* for the Hys; the DMSO (0.05% final concentration) and double distilled water (ddH_2_O) were used as solvent controls. Vinblastine sulfate (Merck KGaA, Darmstadt, Germany) was used as positive control. After 24 h, 48 h and 72 h of treatment, the medium was removed, MTT solution (0.5 mg/mL) was added to the cells and they were incubated for 3 h in dark conditions at 37 °C. DMSO was used to solubilize the formazan crystals and the absorbance was read by Tecan Sunrise™ (Tecan Group Ltd., Männedorf, Switzerland) UV-vis spectrophotometer at 595 nm. The obtained optical density values were converted into percentage of cell viability and the data were elaborated with AAT Bioquest EC_50_ Calculator (Sunnyvale, CA, USA) [19] in order to obtain the concentration at which the investigated substance exerts half of its maximal response values (EC_50_). The values were repeated three times and reported as mean ± SD.

### 2.6. Antibacterial Activity

To delineate the antibacterial profiles of *R. officinalis* and *L. angustifolia* EOs and Hys, the MIC (minimal inhibitory concentration), the MBC (minimal bactericidal concentration), the agar disk-diffusion method and the VPT (vapor phase test) were used. Five different bacteria strains were considered for this study: *Escherichia coli* ATCC^®^ 25922, *Pseudomonas fluorescens* ATCC^®^ 13525 and *Acinetobacter bohemicus* DSM 102855 as Gram-negative bacteria, and *Kocuria marina* DSM 16420 and *Bacillus cereus* ATCC^®^ 10876 as Gram-positive bacteria. After 24 h of culturing in lysogeny broth (LB) agar, at 26 (for *P. fluorescens*, *A. bohemicus* and *B. cereus*) and 37 °C (for *K. marina* and *E. coli*), the bacterial strains were collected and used for antimicrobial assays.

#### 2.6.1. Minimum Inhibitory Concentration (MIC) and Minimum Bactericidal Concentration (MBC)

The microwell dilution method was carried out to take over the minimum inhibitory concentration (MIC) of the EOs and their corresponding Hys. Twelve two-fold dilutions from 6.25% to 3 × 10^−3^% *v*/*v* and from 50% to 1 × 10^−1^% *v*/*v* for EOs and Hys, respectively, were used. Gentamicin was used as a positive control and the negative and solvent controls were also added. A total of 10^6^ colony-forming unit (CFU)/mL of bacteria in LB broth was seeded in 96-well plates, and after 24 h of incubation with the treatments the bacterial growth was evaluated by adding 10 µL of MTT (200 µg/mL) to each well [36,37]. Before adding MTT in the microwell plates, 10 µL of the last four dilutions without bacterial growth were taken and seeded on LB agar Petri plates to evaluate the minimum bactericidal concentration (MBC) or the concentrations for which no bacterial growth was observed after 24 h of incubation. Furthermore, the MBC/MIC ratios were reported and the values >4 defined bacteriostatic activity, while the ratio MBC/MIC ≤4 defined bactericidal activity for the tested samples [38]. All the assays were carried out in triplicate.

#### 2.6.2. Agar Disk-Diffusion Method and Vapor Phase Test (VPT)

The agar disk-diffusion method was carried out to study the activity of the liquid phase. A total of 10^8^ CFU/mL of bacteria was seeded on a Petri dish with LB agar and 10 µL of pure EOs and 15 µL of Hys. Gentamicin (10 mg/mL) was used as a positive control. After 24 h of incubation at the corresponding temperature, the diameter of the growth inhibition halo or inhibition zone (IZ) was measured by a vernier caliper rule [36]. To evaluate the antibacterial activity of the vapor phase of the EOs and Hys, the vapor phase test (VPT) was used. The assay was performed by seeding 10^8^ CFU/mL of bacteria in a Petri dish with LB agar and pouring 5 mL of LB agar in the Petri plate cover where 6 mm sterile disks had been placed and soaked with 10 and 15 µL of the EOs and the corresponding Hys, respectively. The Petri plates were sealed with parafilm in order to prevent any vapor leakage. After 24 h of incubation, the inhibition halo was measured by a vernier caliper rule [33]. The means and standard deviations were obtained by triplicate measures of the Agar disk-diffusion and VPT halos. 

### 2.7. Antioxidant Activity

#### 2.7.1. DPPH Scavenging Activity Assay

Using the protocol described by Sanchez-Moreno et al. [39], the antioxidant activity of *R. officinalis* and *L. angustifolia* EOs and Hys was assayed by exploiting the 1,1-diphenyl-2-picrilidrazil radical (DPPH•) properties in the DPPH scavenging activity assay. A total of 100 µL of twelve concentrations obtained by geometric dilutions of each sample (from 25% to 0.01%) were added to a 96-well plate and mixed with 100 µL of a solid DPPH methanol solution (0.2 mM). EOs and Hys without DPPH solution were added as controls and Trolox dilutions and DPPH plus methanol were also added. After 30 min of incubation, the absorbances were measured by a Tecan Sunrise™ UV-vis spectrophotometer (517 nm). Three replicates of the experiment were carried out.

#### 2.7.2. ABTS Radical Scavenging Assay

The ABTS (2,2′-azino-bis(3-ethylbenzothiazoline-6-sulfonic acid) diammonium salt) assay was used to investigate the radical scavenging activity [40]. An aqueous 7 mM solution of ABTS was mixed with K_2_S_2_O_8_ (140 mM) and incubated for 16 h, protected from light at room temperature to allow compounds to form the ABTS+• radical cation. The obtained solution was then diluted in ethanol until the absorbance of 0.70 ± 0.02 at 734 nm was reached. Five geometrical dilutions of each sample were prepared and 20 µL was added to 980 µL of the ABTS+• solution (starting from 2% to 0.125%). After 5 min of incubation, the absorbances of the resulting solutions were measured using a Jasco (Jasco Corporation, Lecco, Italy) V-630 UV-Visible spectrophotometer at 734 nm by the Spectra Manager™ software (version II). The blank consisted of a solution composed of 20 µL of geometrical dilutions of EOs and Hys and 980 µL of ethanol. Trolox geometrical dilutions were used as a positive control. Data were collected in triplicate.

### 2.8. IC50 and Trolox Equivalents

Using the following equation, the percentage of antioxidant activity (*AA*%) was estimated:

(1) $$AA\%=\left[\frac{A_{blank}-A_{sample}}{A_{blank}}\right]\times \;100$$

The percentage of antioxidant activity (*AA*%) (*y*) was then plotted against the sample concentrations (*x*) to form a regression line (*y* = *mx* + *q*). The *IC*_50_ value was calculated by the following formula:

(2) $$IC_{50}=\frac{50-q}{m}$$

The Trolox equivalent antioxidant capacity (TEAC) was also used to express the antioxidant capacity and expressed in µmol Trolox/mg of EOs or Hys.

### 2.9. Statistical Analysis

The Friedman test, a well-known distribution-free test for the randomized blocks analysis of variance [41], was used for investigating homogeneity in the cytotoxic activity of EOs and Hys across time. Furthermore, for both cytotoxic and antibacterial activities, statistical discrepancies between *R. officinalis* and *L. angustifolia* were evaluated by adopting the Mann–Whitney–Wilcoxon nonparametric test [41]. All the statistical tests were performed using R Statistical Software version 4.1.0 (The R foundation for Statistical Computing, Vienna, Austria).

## 3. Results

### 3.1. Liquid and Vapor Phase EOs Chemical Volatile Composition

GC–MS and HS/GC–MS techniques were used to investigate the liquid- and vapor-phase chemical composition of *R. officinalis* and *L. angustifolia* EOs and Hys. The composition of *R. officinalis* EO in Table 1 is reported. Monoterpene hydrocarbons (71.5% and 89.5%) were the main compounds followed by oxygenated monoterpenes (26.0% and 10.5%) in the liquid and vapor phase, respectively. Among them, α-pinene (51.2%; 74.7%) and 1,8-cineole (20.1%; 10.0%) were the most abundant components in both phases. Sesquiterpene compounds such as β-caryophyllene (1.4%), α-curcumene (0.1%) and caryophyllene oxide (0.1%) were detected only in the liquid phase.

Twenty-five volatile compounds were identified in *L. angustifolia* EO and they are listed in Table 2. In this EO, oxygenated monoterpenes prevailed over monoterpene hydrocarbons with relative percentages equal to 86.9% in the liquid phase and 53.7% in the vapor phase. Linalool (49.9% and 26.2%) was the major compound both in the liquid and vapor phase, respectively. The second most abundant compound was linalyl acetate (17.9%) in the liquid phase while α-pinene (17.8%) was in the vapor phase. Additionally, in this case, sesquiterpenes were present only in the liquid phase with β-caryophyllene (1.5%) and α-farnesene (1.2%) as main components.

### 3.2. Vapor Phase Hys Chemical Composition

The volatile chemically identified compounds of Hys vapor phase are listed in Table 3. They were only composed of oxygenated monoterpenes, and 1,8-cineole (56.2%) and linalool (42.9%) were the major components of *R. officinalis* and *L. officinalis* Hys, respectively. Camphor (20.3% and 18.4%) was the second major component with similar percentage values in both Hys. 

**Figure 1:**
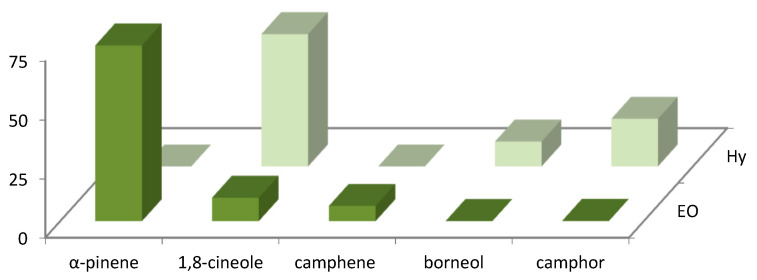
Bar graph of main compounds detected in vapor-phase *R. officinalis* Hy and EO.

**Figure 2:**
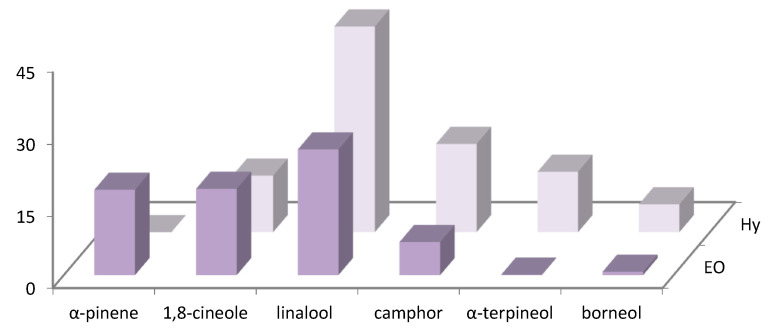
Bar graph of main compounds detected in vapor-phase *L. angustifolia* Hy and EO.

The distribution of the main volatile compounds detected in *R. officinalis* and *L. officinalis* Hys and in the vapor-phase EOs is shown in Figure 1 and Figure 2.

### 3.3. Cytotoxic Activity

**Figure 3:**
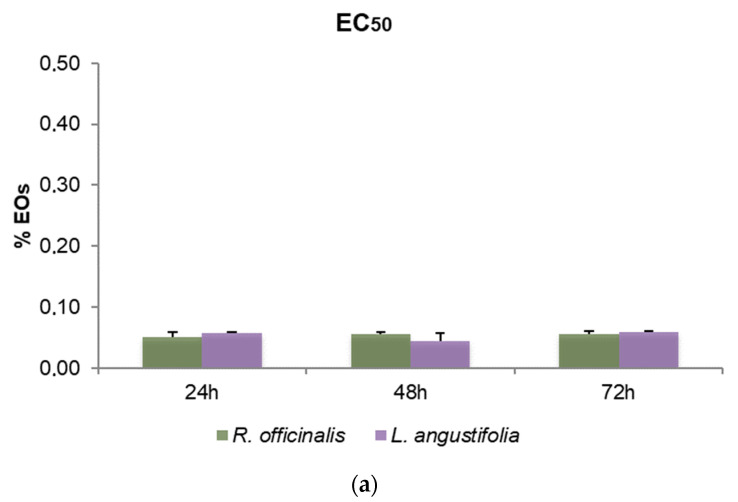

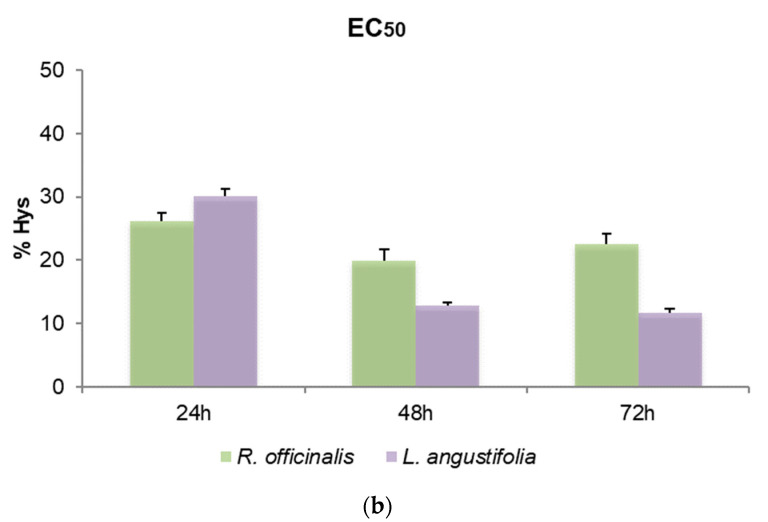
Bar graphs of EC_50_ values obtained by thiazolyl blue tetrazolium bromide assay (MTT) after 24, 48 and 72 h of SHSY5Y cell treatments, (**a**) with *R. officinalis* and *L. angustifolia* EOs and (**b**) with the corresponding Hys. Error bars: standard deviation.

To define the cytotoxic effects of *R. officinalis* and *L. angustifolia* EOs and their corresponding Hys, MTT assays were carried out and the EC_50_ values after 24, 48 and 72 h for SHSY5Y cells were reported (Figure 3). Friedman test *p*-values for assessing the homogeneity of EC_50_ across time with respect to EOs and Hys of both plant species are displayed in Table 4. A very mild significance for Hys (*p*-value equal to 5%) can be detected, while, as highlighted also in Figure 3a, EOs were revealed to be cytotoxic in a time-independent manner, showing EC_50_ values quite similar in the two plant species considered, from 0.05% to 0.06% *v*/*v* for the three time points evaluated (*p*-values higher than 5%). Even if *p*-values related to Hys are equal to 5%, the corresponding hypothesis could be barely rejected due to the rather small sample size. Consequently, the Hys EC_50_ values may be considered substantially stable for each time point and plant species. *R. officinalis* Hys was slightly lower at 24 h than the *L. angustifolia* Hys (26.82 ± 2.39% and 30.18 ± 1.11%, respectively) while the latter was more active (19.96 ± 4.7% and 12.78 ± 0.58%) after 48 h and after 72 h (21.53 ± 3.28% and 11.72 ± 0.60%), respectively (Figure 3b). An EC_50_ value of 1.94 ± 0.18 nM for vinblastine treatment was obtained. DMSO control did not affect cell viability.

Owing to the effective homogeneity resulting from the Friedman test, the data were reduced to the means of EC_50_ measurements across time. Therefore, in order to assess the EC_50_ values homogeneity between *R. officinalis* and *L. angustifolia*, two Mann–Whitney–Wilcoxon tests were implemented. From the obtained results (both *p*-values equal to 1), *R. officinalis* and *L. angustifolia* produced statistically equivalent EC_50_ values both for EOs and Hys.

### 3.4. Antibacterial Activity

Investigations on *R. officinalis* and *L. angustifolia* EOs and Hys antibacterial activity were executed by different assays. In Table 5, the results obtained by *R. officinalis* EO are listed. The highest antibacterial activity was against *A. bohemicus* with an MIC value of 0.19%, MBC value of 0.39%, IZ of 9.17 ± 0.76 mm and VIZ of 80.00 ± 00 mm. Concerning the other tested bacterial strains, MIC values ranged from 0.39% to 3.13% and MBC values from 0.39% to 6.25%. The MIC/MBC ratio showed that the tested EOs possessed bactericidal properties. IZs were 7.33 ± 0.58 and 8.33 ± 1.52 mm for *K. marina* and *B. cereus*, respectively, and VIZs were 80.00 ± 00 mm. For *E. coli*, IZ was 7.00 ± 0.00 mm, while the EO vapor phase did not determine bacterial growth inhibition. *R. officinalis* Hy was not active against growth of the tested bacteria (Table 5). 

*L. angustifolia* EO presented a high antibacterial activity with MIC values of 0.39%, 1.56%, 0.19%, 0.78% and 0.19% and MBC values of 0.39%, 3.13%, 0.39%, 0.78% and 0.19% for *E. coli, P. fluorescens, A. bohemicus, K. marina* and *B. cereus*, respectively. MIC/MBC ratios showed that the tested EO possessed bactericidal properties. IZ values for *R. officinalis* Hy were 11.00 ± 1.00, 7.17 ± 0.76, 11.67 ± 1.15, 11.33 ± 1.53 and 10.67 ± 0.58 mm against *E. coli, P. fluorescens, A. bohemicus, K. marina* and *B. cereus.* VIZ values were 6.17 ± 1.04 and 0.67 ± 1.15 mm against *A. bohemicus* and *B. cereus*, respectively. *L. angustifolia* Hy was not effective against the tested bacterial strains growth (Table 6).

IZ and VIZ values for *R. officinalis* and *L. angustifolia* EOs were considered for assessing a significant discrepancy in the antibacterial activities. Mann–Whitney–Wilcoxon p-values suggest that, for any significance level greater than 5%, the antibacterial activity against *E. coli*, *A. bohemicus* and *K. marina*, in terms of IZ values, is greater for *L. angustifolia* EO than for *R. officinalis* EO (exact p-values equal to 5%), while this cannot be concluded for the antibacterial activity against *B. cereus* (exact *p*-value equal to 10%). Contrarily, when VIZ values are considered, the Mann–Whitney–Wilcoxon p-values suggest that for any significance level greater than 5%, the antibacterial activity against *A. bohemicus* and *B. cereus* is greater for *R. officinalis* EO than for *L. angustifolia* EO (exact *p*-values equal to 5%).

### 3.5. Antioxidant Activity of R. officinalis and L. angustifolia EOs and Hys

The antioxidant potential of the EOs and the Hys was investigated by DPPH and ABTS assays reporting the IC_50_ values (Table 7). Results are also expressed in µM Trolox/mg of samples. The highest antioxidant activity was measured in the EOs followed by Hys of both plants. For *R. officinalis* EO, IC_50_ was 13.48 ± 1.58 and 20.21 ± 2.72 µg/mL, while for *L. angustifolia* EO IC_50_ was 7.75 ± 0.10 and 18.71 ± 2.15 µg/mL for DPPH and ABTS, respectively. TEAC values were 1.90 ± 1.14 and 23.53 ± 2.43 µmol/mg for *R. officinalis* EO and 3.30 ± 0.09 and 25.45 ± 3.72 µmol/mg for *L. angustifolia* EO, for DPPH and ABTS, respectively. *R. officinalis* and *L. angustifolia* Hys showed antioxidant activity lower than the corresponding EOs. In particular, *R. officinalis* Hy IC_50_ was 136.30 ± 3.85 and 349.42 ± 19.32 µg/mL and TEAC values were 0.22 ± 0.02 and 1.35 ± 0.03 µmol/mg by DPPH and ABTS, respectively. On the contrary, *L. angustifolia* Hy antioxidant activity was slightly lower than *R. officinalis* Hy in DPPH assay (240.02 ± 13.65 µg/mL and 0.12 ± 0.01 µmol/mg) and higher in ABTS assay (181.24 ± 15.71 µg/mL and 2.62 ± 0.30 µmol/mg, for IC_50_ and TEAC, respectively).

## 4. Discussion

In our investigation, liquid and vapor phases of *L. angustifolia* EO were characterized by linalool (49.9% and 26.2%) as a major constituent, while *R. officinalis* EO was characterized by α-pinene (51.2% and 74.7%). The chemical profile of *L. angustifolia* and *R. officinalis* EOs obtained by aerial parts of plants growing in Syria was investigated by Al-Younis et al. [42], showing a similar composition with borneol (16.25%) and linalool (35.12%) as the main components, respectively, whereas *L. angustifolia* EO collected in Xinjiang [43] exhibited linalyl acetate (28.89%) as a principal molecule, as well as the Himalayan one (47.56%) [44]. Reagrding *R. officinalis* EO, 1,8-cineole was the most abundant component in EO from China [45] and Belgrade [46] (26.54% and 43.77%, respectively). On the other side, α-pinene (43.9%) and p-cymene (44.02%) were found to be those with the higher percentage in *R. officinalis* EO from Iran [47] and Turkey [48]. The present study is the first on the characterization of the aroma profiles of *R. officinalis* and *L. angustifolia* Hys grown in the Tuscany region using HS/GC–MS. The obtained results highlight that they were characterized exclusively by oxygenated monoterpenes, among which 1,8-cineole (56.2% in *R. officinalis* Hy) and linalool (42.9% in *L. angustifolia* Hy) were the major exponents. Previous studies showed a different chemical composition, with camphor (24.9%), terpinen-4-ol (51.9%) and verbenone (45.31%) as the main components of the *R. officinalis* hydrolates from Japan [49], Colombia [50] and Korea [51], respectively. Regarding *L. angustifolia* Hy, a similar composition to that described by us was reported for extracts from Poland [27,52], in which linalool (24.6% and 26.5%, respectively) was also the major detected compound. Of interest, *L. angustifolia* Hy from Croatia [53] was characterized by linalool (23.2%) when steam distillation was used to obtain the extract, and by 1,8-cineole (20.6%) through hydrodistillation. In fact, the chemical profile of Hys can also vary according to the distillation method used [54].

Our findings and the cited references confirm that the variability of the chemical composition of EOs and Hys depends on various exogenous and endogenous factors, such as the area of provenance of the plant and the extraction method [55].

The biological properties of the *R. officinalis* and *L. angustifolia* EOs were investigated with in vivo and in vitro models [9,56] and numerous studies demonstrated their exertion of biological activities [57]. In the present study, *R. officinalis* and *L. angustifolia* EOs and Hys, cytotoxic, antimicrobial and antioxidant activities were investigated. Both EOs exerted cytotoxic activities with low EC_50_ values, corresponding with a high inhibition of SHSY5Y cell proliferation. Regarding Hys treatments, higher EC_50_ values were obtained and *L. angustifolia* Hy IC_50_ was slightly lower than *R. officinalis* Hy at 48 and 72 h. Studies on Hys cytotoxicity and antiproliferative effects are limited. *Melissa officinalis, Achillea teretifolia, Achillea aleppica, Origanum onites* and *Salvia fruticosa* have been investigated on a colorectal cancer cell line to determine their cytotoxic and cytostatic effects, and *O. onites* Hy was the most effective [58].

In this paper, DPPH and ABTS assays demonstrated that the EOs and Hys possess antioxidant activities, although showing different IC_50_ values. Antibacterial properties of the Hys were investigated and, unlike EOs, they were not able to inhibit bacterial growth in the different assays. 

As reviewed by D’Amato et al. [31], the antibacterial and antifungal, as well antioxidant, properties of the Hys were demonstrated for different plant species, and their uses and applications could be evaluated to control microorganism growth and oxidative processes in food shelf-life. Furthermore, Hys were demonstrated to be active against biofilm production [59,60], and thus could be used as a natural antimicrobial agent for food production [54]. As reported by Šilha et al. [53], no antibacterial effect of *L. angustifolia* Hy was detected against eight strains of *Arcobacter*-like bacteria and against *Staphylococcus aureus*, *Enterococcus faecalis*, *Pseudomonas aeruginosa*, *Escherichia coli* and the yeast *Candida albicans.* On the contrary, concentrated Hys, obtained by solid-phase extraction and tested against the same microorganisms, exercised a considerable antimicrobial activity. 

On the other side, *R. officinalis* EO showed antioxidant and antimicrobial properties while its corresponding Hy had neither a potential effect against *E. coli*, *P. aeruginosa*, *S. aureus* and *C. albicans* and *A. niger*, nor antioxidant properties [50].

Notably, lavandin (*L. x intermedia*) Hy was not reported to be active against *E. coli* and *B. cereus*, whereas it showed antibacterial activity against the same bacterial strains when formulated in nanoemulsion [61].

Hys being composed of the condensed water of the distillation process and by only some volatile oil components, their chemical composition is different with respect to the corresponding EOs; however, the amounts of the main components can vary greatly [31,62]. Generally, Hys exert their biological activities at high concentrations, reflecting their low terpene amount. Since they are aqueous solutions, the hydrophilic environment likely facilitates the terpenes’ availability, enhancing their biological activity [54].

## 5. Conclusions

Chemical investigations performed by HS/GC–MS revealed that EOs and Hys of *R. officinalis* and *L. angustifolia* are rich in bioactive compounds. Both EOs revealed a good antibacterial and antioxidant activity, while their respective Hys exerted a slight antioxidant activity and were completely inactive on the selected bacterial strains. The antiproliferative activity was also evaluated by highlighting, for the first time, that not only EOs but also Hys exerted a cytotoxic effect.

In conclusion, *R. officinalis* and *L. angustifolia* EOs, thanks to their exhibited biological activities, could have potential applications in various fields, including foods and beverages. It is also interesting to note that the results obtained with the Hys on the SHSY5Y cell line underline the potentiality of these by-products of the distillation process. In this regard, our findings will be useful for further studies and applications.

## Figures and Tables

**Table 1:** Chemical composition (%) of liquid and vapor phase *R. officinalis* essential oil (EO).

N°	COMPONENT ^1^	LRI ^2^	LRI ^3^	*R. officinalis* (%) ^4^	*R. officinalis* (%) ^5^
1	α-pinene	945	943	51.2 ± 0.02	74.7 ± 0.1
2	camphene	948	946	5.6 ± 0.04	6.5 ± 0.02
3	dehydrosabinene	961	958	1.1 ± 0.04	1.1 ± 0.02
4	β-myrcene	982	983	2.0 ± 0.05	1.1 ± 0.02
5	β-pinene	990	986	3.4 ± 0.03	2.8 ± 0.02
6	α-phellandrene	1000	1006 *	0.2 ± 0.02	-
7	α-terpinene	1010	1008	0.4 ± 0.02	0.2 ± 0.02
8	p-cymene	1020	1016	2.0 ± 0.05	0.9 ± 0.02
9	limonene	1022	1023	4.8 ± 0.03	1.7 ± 0.02
10	1,8-cineole	1025	1027	20.1 ± 0.15	10.0 ± 0.05
11	γ-terpinene	1051	1054	0.9 ± 0.04	0.3 ± 0.02
12	terpinolene	1080	1078	0.7 ± 0.02	0.2 ± 0.02
13	p-cymenene	1085	1083.4	0.1 ± 0.01	-
14	linalool	1096	1092	1.0 ± 0.05	0.2 ± 0.02
15	camphor	1125	1126	0.8 ± 0.06	0.2 ± 0.04
16	borneol	1155	1152	0.2 ± 0.02	0.1 ± 0.01
17	endo-borneol	1158	1155	0.9 ± 0.03	-
18	terpinen-4-ol	1161	1160	0.3 ± 0.02	-
19	α-terpineol	1182	1183	0.4 ± 0.01	-
20	verbenone	1192	1196	0.6 ± 0.02	-
21	geraniol	1234	1237	0.4 ± 0.02	-
22	bornyl acetate	1262	1268	1.0 ± 0.07	-
23	nerol acetate	1362	1363	0.3 ± 0.02	-
24	β-caryophyllene	1424	1426	1.4 ± 0.03	-
25	α-curcumene	1478	1475	0.1 ± 0.02	-
26	caryophyllene oxide	1586	1583	0.1	-
	SUM (%)			100.0	100.0
	Monoterpene hydrocarbons			71.5	89.5
	Oxygenated monoterpenes			26.0	10.5
	Sesquiterpene hydrocarbons			1.5	-
	Oxygenated sesquiterpene			0.1	-
	Others			-	-

^1^ The components are reported according to their elution order on a polar column; ^2^ linear retention indices measured on a polar column; ^3^ linear retention indices from literature; ^4^ percentage mean values of *R. officinalis* EO components (liquid phase); ^5^ percentage mean values ± SD (standard deviation) of *R. officinalis* EO components (vapor phase); * Normal Alkane retention index; -, not detected.

**Table 2:** Chemical composition (%) of liquid and vapor phase *L. angustifolia* EO.

N°	COMPONENT ^1^	LRI ^2^	LRI ^3^	*L. angustifolia* (%) ^4^	*L. angustifolia* (%) ^5^
1	α-pinene	945	943	1.6 ± 0.02	17.8 ± 0.02
2	camphene	948	946	0.8 ± 0.02	8.9 ± 0.05
3	β-pinene	990	986	2.3 ± 0.02	1.2 ± 0.02
4	α-phellandrene	1000	1006 *	0.1 ± 0.02	0.5 ± 0.05
5	α-terpinene	1010	1008	0.2 ± 0.02	0.5 ± 0.03
6	p-cymene	1020	1016	0.1 ± 0.02	1.0 ± 0.02
7	limonene	1022	1023	1.2 ± 0.02	6.2 ± 0.02
8	1,8-cineole	1025	1027	5.7 ± 0.02	18.0 ± 0.03
9	cis-β-ocimene	1033	1032	0.1 ± 0.02	3.2 ± 0.05
10	trans-β-ocimene	1041	1043	1.4 ± 0.02	3.5 ± 0.03
11	γ-terpinene	1051	1054	0.6 ± 0.02	2.3 ± 0.03
12	linalol oxide	1073	1073	0.5 ± 0.02	0.2 ± 0.03
13	terpinolene	1080	1078	0.6 ± 0.01	1.1 ± 0.03
14	linalool	1096	1092	49.9 ± 0.14	26.2 ± 0.05
15	camphor	1125	1126	3.2 ± 0.04	6.9 ± 0.05
16	borneol	1155	1152	3.9 ± 0.02	0.7 ± 0.02
17	terpinen-4-ol	1161	1160	5.0 ± 0.05	1.4 ± 0.02
18	α-terpineol	1182	1183	0.8 ± 0.04	0.1 ± 0.02
19	linalyl acetate	1251	1252	17.9 ± 0.02	0.2 ± 0.02
20	β-caryophyllene	1424	1426	1.5 ± 0.02	-
21	cis-β-farnesene	1444	1441	0.8 ± 0.01	-
22	β-bisabolene	1500	1501	0.3 ± 0.02	-
23	α-farnesene	1505	1506	1.2 ± 0.02	-
24	caryophyllene oxide	1586	1583	tr	-
25	α-bisabolol	1662	1665	0.3 ± 0.02	-
	SUM (%)			100.0	99.9
	Monoterpene hydrocarbons			9.0	46.2
	Oxygenated monoterpenes			86.9	53.7
	Sesquiterpene hydrocarbons			3.8	-
	Oxygenated sesquiterpene			0.3	-
	Others			-	-

^1^ The components are reported according to their elution order on a polar column; ^2^ linear retention indices measured on a polar column; ^3^ linear retention indices from literature; ^4^ percentage mean values of *L. officinalis* EO components (liquid phase); ^5^ percentage mean values ± SD (standard deviation) of *L. officinalis* EO components (vapor phase); * Normal Alkane retention index; -, not detected; tr, trace < 0.1.

**Table 3:** Chemical composition (%) of vapor-phase *R. officinalis and L. angustifolia* Hys.

N°	COMPONENT ^1^	LRI ^2^	LRI ^3^	*R. officinalis* (%) ^4^	*L. angustifolia* (%) ^5^
1	1,8-cineole	1025	1027	56.2 ± 0.04	11.8 ± 0.03
2	linalol oxide	1073	1073	-	0.1 ± 0.01
3	linalool	1096	1092	4.2 ± 0.02	42.9 ± 0.05
4	camphor	1125	1126	20.3 ± 0.02	18.4 ± 0.02
5	borneol	1155	1152	10.6 ± 0.02	5.8 ± 0.02
6	terpinen-4-ol	1161	1160	1.6 ± 0.02	8.4 ± 0.02
7	α-terpineol	1182	1183	2.0 ± 0.04	12.6 ± 0.02
8	verbenone	1192	1196	5.1 ± 0.09	-
	SUM (%)			100.0	100.0
	Monoterpene hydrocarbons			-	-
	Oxygenated monoterpenes			100.0	100.0
	Sesquiterpene hydrocarbons			-	-
	Oxygenated sesquiterpene			-	-
	Others			-	-

^1^ The components are reported according to their elution order on a polar column; ^2^ linear retention indices measured on a polar column; ^3^ linear retention indices from literature; ^4^ percentage mean values ± SD (standard deviation) of *R.* *officinalis* Hydrolate (Hy) components; ^5^ percentage mean values of *L. officinalis* Hy components; -, not detected.

**Table 4:** Friedman test *p*-values for testing homogeneity across time.

Sample	*p*-Value
*R. officinalis* EOs	0.717
*L. angustifolia* EOs	0.097
*R. officinalis* Hys	0.050
*L. angustifolia* Hys	0.050

**Table 5:** Antibacterial activity of *Rosmarinus officinalis* EO and Hy.

	*R. officinalis* EO					*R. officinalis* Hy				
	MIC ^1^	MBC ^2^	MBC/MIC Ratio	IZ ^3^	VIZ ^4^	MIC ^1^	MBC ^2^	MBC/MIC Ratio	IZ ^3^	VIZ ^4^
*E. coli*	3.13	3.13	1.00	7.00 ± 0.00	-	na	na	-	-	-
*P. fluorescens*	3.13	6.25	0.50	-	-	na	na	-	-	-
*A. bohemicus*	0.19	0.39	0.50	9.17 ± 0.76	80 ± 00	na	na	-	-	-
*K. marina*	1.56	3.13	0.50	7.33 ± 0.58	80 ± 00	na	na	-	-	-
*B. cereus*	0.39	0.39	1.00	8.33 ± 1.52	80 ± 00	na	na	-	-	-

^1^ Minimal inhibitory concentration (% *v*/*v*) of *R. officinalis* EO and HY; ^2^ minimal bactericidal concentration (% *v*/*v*) of EO and Hy; ^3^ growth inhibition zone by disc diffusion assay (mm); ^4^ growth inhibition zone by vapor phase test (mm); na, not attained; -, not detected. Values are expressed as means ± SD. Gentamicin determined MIC and MBC values of 6.25 µg/mL for *E. coli*, 3.13 µg/mL for *P. fluorescens,* 6.25 µg/mL for *A. bohemicus,* 1.56 µg/mL for *K. marina* and 1.56 µg/mL for *B. cereus* and IZ values of 17.00 ± 1 mm for *E. coli*, 19.67 ± 0.58 mm for *P. fluorescens*, 24.33 ± 1.53 mm for *A. bohemicus*, 24.67 ± 1.53 mm for *K. marina* and 19.67 ± 1.53 mm for *B. cereus*.

**Table 6:** Antibacterial activity of *L. angustifolia* EO and Hy.

	*L. angustifolia* EO					*L. angustifolia* Hy				
	MIC ^1^	MBC ^2^	MBC/MIC Ratio	IZ ^3^	VIZ ^4^	MIC ^1^	MBC ^2^	MBC/MIC Ratio	IZ ^3^	VIZ ^4^
*E. coli*	0.39	0.39	1.00	11.00 ± 1.00	-	na	na	-	-	-
*P. fluorescens*	1.56	3.13	0.50	7.17 ± 0.76	-	na	na	-	-	-
*A. bohemicus*	0.19	0.39	0.50	11.67 ± 1.15	6.17 ± 1.04	na	na	-	-	-
*K. marina*	0.78	0.78	1.00	11.33 ± 1.53	-	na	na	-	-	-
*B. cereus*	0.19	0.19	1.00	10.67 ± 0.58	0.67 ± 1.15	na	na	-	-	-

^1^ Minimal inhibitory concentration (% *v*/*v*) of *L. angustifolia* EO and HY; ^2^ minimal bactericidal concentration (% *v*/*v*) of EO and Hy; ^3^ growth inhibition zone by disc diffusion assay (mm); ^4^ growth inhibition zone by vapor phase test (mm); na, not attained; -, not detected. Values are expressed as means ± SD. Gentamicin determined MIC and MBC values of 6.25 µg/mL for *E. coli*, 3.13 µg/mL for *P. fluorescens,* 6.25 µg/mL for *A. bohemicus,* 1.56 µg/mL for *K. marina* and 1.56 µg/mL for *B. cereus* and IZ values of 17.00 ± 1 mm for *E. coli*, 19.67 ± 0.58 mm for *P. fluorescens*, 24.33 ± 1.53 mm for *A. bohemicus*, 24.67 ± 1.53 mm for *K. marina* and 19.67 ± 1.53 mm for *B. cereus*.

**Table 7:** Antioxidant activity of *R. officinalis* and *L. angustifolia* EOs and Hys.

		*R. officinalis* EO	*R. officinalis* Hy	*L. angustifolia* EO	*L. angustifolia* Hy
**DPPH**	**IC_50_** *	13.48 ± 1.59	136.30 ± 3.85	7.75 ± 0.10	240.02 ± 13.65
	**TEAC** **	1.90 ± 0.15	0.22 ± 0.03	3.30 ± 0.10	0.12 ± 0.01
**ABTS•+**	**IC_50_** *	20.20 ± 2.72	349.42 ± 19.32	18.71 ± 2.16	181.24 ±15.71
	TEAC **	23.53 ± 2.43	1.35 ± 0.03	25.45 ± 3.73	2.62 ± 0.31

* µg/mL; ** µmol/mg. Values are expressed as means ± SD; half maximal inhibitory concentration (IC_50_); 2,2′-azino-bis (3-ethylbenzothiazoline-6-sulfonic acid) diammonium salt based assay (ABTS•+); 2,2-diphenyl-1-picrylhydrazyl assay (DPPH); Trolox equivalent antioxidant capacity (TEAC).

## Data Availability

All generated data are included in this article.
